# One-Year Phenology of Leaf Gas Exchange Dynamics in *Coccocypselum lanceolatum*

**DOI:** 10.3390/biology15130994

**Published:** 2026-06-24

**Authors:** Miroslava Rakocevic

**Affiliations:** 1 Setor de Fisiologia Vegetal, Laboratório de Melhoramento Genético Vegetal, Centro de Ciências e Tecnologias Agropecuárias, Universidade Estadual do Norte Fluminense (UENF), Avenida Alberto Lamego 2000, Parque California, Campos dos Goytacazes 28013-602, RJ, Brazil; mimarako@unicamp.br; Tel.: +55-19-97161-8918; 2 Laboratory of Crop Physiology, Department of Plant Biology, Institute of Biology, State University of Campinas (UNICAMP), Campinas 13083-862, SP, Brazil

**Keywords:** dark respiration, herbaceous species, iWUE, leaf assimilation, leaf transpiration, light compensation point, PPFD, R:FR, Rubiaceae, stomatal conductance

## Abstract

*Coccocypselum lanceolatum* is a tropical, perennial, creeping, herbaceous C3 plant species that is found in deeply shaded humid forests. This species has potential for medicinal and culinary uses. Knowledge about this species and other herbaceous Rubiaceae is confined to phytocoenological and morpho-anatomical studies. Here, it was hypothesized that (1) leaf gas exchange dynamics over a one-year period in *C. lanceolatum* are related to light conditions, phenology and environmental seasonal changes; (2) photosynthetic performance is focused on enhanced carbon gains through a high leaf net assimilation rate (*A*_net_) relative to light availability, a low dark respiration rate (*R*_d_) and a light compensation point (LCP); and (3) these parameters will vary over leaf age. The photosynthetic photon flux density (PPFD), characterizing the growth and development of *C. lanceolatum*, was reduced to 4–11% of incoming light in the open area, while the red-to-far-red light ratio (R:FR) was reduced from 1.15 to mean diurnal values of 0.45–0.81, depending on forest canopy dynamics. Leaf gas exchange parameters [*A*_net_, stomatal conductance (*g_s_*), leaf transpiration (*E*), and intrinsic water use efficiency (iWUE)] were observed over a one-year period. *A*_net_, *g_s_*, and *E* were correlated with energy factors (PPFD and air temperature) during vegetative growth, while only iWUE showed a correlation with leaf gas exchange parameters during blooming and fruiting, indicating that seasonality and phenology were additional drivers of leaf gas exchange. As a deep-shade forest species, *C. lanceolatum* displayed low iWUE (3–21 μmol m^−2^ s^−1^) and was adapted to maximize carbon gain and prioritize high *g_s_* rather than water economy. The extremely low LCP (4.2 μmol m^−2^ s^−1^), low *R*_d_ (0.2 to 0.43 μmol m^−2^ s^−1^), maximum net photosynthesis (*A*_max_, 5 μmol m^−2^ s^−1^), and apparent quantum efficiency of CO_2_ assimilation (Φ of 0.04 µmol µmol^−1^) were adaptational traits of this species for low light. Finally, the *A*_net_, *g_s_*, *E*, iWUE, gross photosynthesis under light saturation, *R*_d_, LCP, and light saturation point values were different when comparing young and adult leaves. The ecophysiological responses over a one-year period shown here could assist in the success of *C. lanceolatum* as a sustainable soil-cover plant in shaded areas.

## Simple Summary

*Coccocypselum lanceolatum* is a tropical, perennial, herbaceous species found in areas of deeply shaded humid forests. This species has potential for medicinal and culinary uses. Knowledge of this plant is predominantly oriented to phytocoenological and morpho-anatomical studies. The ecophysiological parameters, such as leaf photosynthesis, transpiration, water use efficiency, and photosynthetic performance, were investigated to understand these dynamics over a one-year period in relation to variables such as light and temperature. Ecophysiological responses were strongly associated with primary energy variables during the vegetative growth phase, while during flowering and fruiting, correlations were expressed at a lower rate, indicating that seasonality and phenology were additional drivers of leaf gas exchange dynamics. The ecophysiological responses over the one-year period shown here could provide important information about this plant’s successful use as a sustainable soil-cover plant for shaded areas.

## 1. Introduction

*Coccocypselum lanceolatum* (Ruiz & Pavon) Persoon, known as cauabori or blue fruit in Brazil, is a perennial, small creeping herb (usually with short stems of 15–20 cm) from the Rubiaceae family that prefers shady and humid locations [1,2]. The genus *Coccocypselum* comprises 35 neotropical species occurring from Mexico to Argentina. In Brazil, this genus is found in the Caatinga, the Central Brazilian savanna (Cerrado), the Atlantic Rainforest and the Pampas [3]. In southern Brazil (Rio Grande do Sul State), where this experiment was performed, *C. lanceolatum* is usually found in patches of Atlantic forests in mountainous regions [4] and is genetically characterized as 2n = 20, as also found in other species from the Coccocypseleae tribe [5].

Oval pubescent or hirsute *C. lanceolatum* leaves appear in pairs, having a velvety indumentum on both sides and often being hairier in the midrib region [1,2]. *Coccocypselum. lanceolatum* is a C3 hypostomatic species, having stomata predominantly on the abaxial leaf surface [6]. Shoots of this species have colleters, multicellular glands with protective function against dehydration, lubricating young sensitive organs, such as shoot apices, and young leaves [7]. *Coccocypselum lanceolatum* produces pedunculated inflorescences with a reflexed intense blue calyx and ovoid carnose blue berries [8]. Calyx persists at the tip of the berry [1]. This plant species blooms primarily between November and January (although blooming can be observed throughout the year), while fruitification occurs between February and July in the central Brazilian state of São Paulo [9,10]. Blue-colored fruits of wild, unknown berries are often toxic, as are some species of the genus *Vaccinium* [11], while *C. lanceolatum* fruits attract birds [12] and are edible, consumed fresh, or found in jelly, juice, and ice cream.

Rubiaceae is the fourth largest family of flowering plants with a cosmopolitan distribution, predominantly in the tropics, where many species are endemic and ecologically sensitive to environmental fluctuations and climate changes [13]. In this family, about 20% are classified as herbaceous species, while 80% are classified as woody species [14]. Among four groups of environmental variables (water and energy, climate seasonality, historical climate change, and human influence), water and energy are the key factors (followed by climate seasonality) that drive the richness pattern in the Rubiaceae family [14].

The sun is the principal energy and light supplier of our planet. Under low sunlight conditions (for example in shaded forests), common plant responses can be total plant dry matter reduction, as well as reductions in leaf gas exchange parameters, such as the net leaf photosynthetic rate (*A*_net_), dark respiration rate (*R*_d_), leaf transpiration rate (*E*), and stomatal conductance (*g_s_*) [15]. Under forest shade, photosynthetic performance is also modified—it is expressed in reduced maximum net photosynthesis (*A*_max_) and the increased apparent quantum efficiency of CO_2_ assimilation (Φ) in species such as *Ilex paraguariensis* [16]. Shade-adapted adult leaves of *Vitis vinifera* are generally more efficient at using available low light due to the higher chlorophyll content per mass and lower *R*_d_ than young leaves [17]. On the other hand, the shaded young leaves of some *Populus* spp. can show higher *A*_max_, higher maximum stomatal conductance, and higher *R*_d_ than adult leaves, with faster photosynthetic induction and a quicker activation of photoprotective mechanisms when light increases suddenly, during the occurrence of short-term sun flecks [18]. Leaf gas exchange has been widely studied in some Rubiaceae, such as *Coffea* spp. trees [19,20,21], and sporadically in shrubs, such as *Psychotria horizontalis* [22] or *P. limonensis* [23]. For herbaceous understory species of the Rubiaceae family, only *A*_max_ values and the light compensation point (LCP) have been related to *C. lanceolatum* in the group characterization of different vegetation types of Cerrado savanna [24].

Life strategy classification categorizes plants into competitors, stress tolerators, and ruderals, reflecting their adaptations to resource use and disturbance intensity [25]. Stress tolerators survive via the maintenance of metabolic performance in variable and unproductive niches, and they are separated from other classes based on morphophysiological traits rather than morphological traits [26]. Slow-growing stress tolerators have low *A*_net_, *R*_d_, and nitrogen levels [27]. In tropical biomes, understory plants are exposed to continuous shading due to the dense forest canopy [24]. In such habitats, shade tolerance represents the plant’s ability to efficiently modify its morphophysiology in order to cope with shade stress [28].

When plants are shaded by other vegetation, light quality is modified together with light intensity. Red (R) and blue (B) light are absorbed for photosynthesis and thus depleted from the transmitted light, whereas far-red (FR) wavelengths are transmitted and reflected by green tissues [29]. Such modifications are perceived as the red-to-far-red light ratio (R:FR) decreases, which triggers shade-avoidance responses (in shade-avoiders) generally characterized by increased shoot length, decreased branching/tillering and larger, thinner leaves [30]. In plants, different light wavelengths are perceived and signaled through a variety of photoreceptors, such as phytochromes, cryptochromes and phototropins [31]. Light quality modifications regulate the whole life cycle of plants through light receptor conduction. Plants adjust their growth and development by sensing photoperiods adequate for development and phenology with photoreceptors being parallelly involved in plant responses to environmental pressures that occur over seasonal changes [31]. In green tissues of shade avoiders, a low R:FR ratio (660 and 730 nm, respectively) is primarily perceived by phytochrome B [32]. On the other hand, shade-tolerant species developed a differential control of phytochrome-interacting factors (PIFs), having an increased involvement of molecular shade avoidance antagonists and the specific regulation of phytohormone biosynthesis [33]. 

Knowledge of *C. lanceolatum* is predominantly oriented to phytocoenology, morpho-anatomical studies, and recently basic genetic analysis with a gap in knowledge of functional responses and their dynamics, which could assist in future cultivation. It was hypothesized that (1) leaf gas exchange dynamics over a one-year period in *C. lanceolatum* are related to light conditions, phenology and environmental seasonal changes, (2) photosynthetic performance is focused on enhanced carbon gains through high *A*_net_ relative to low-light availability, low *R*_d_ and LCP, and (3) ecophysiological parameters express differences between different leaf age groups. For that, the light quality and intensity inside of the forest, followed by leaf *A*_net_, *g_s_ E* and intrinsic water use efficiency (iWUE), were observed at two-monthly intervals in situ for young and adult leaves, comparing three phenological stages (vegetative, blooming, and fruiting). Additionally, photosynthetic performance was observed during the vegetative stage in both young and adult leaves.

## 2. Materials and Methods

### 2.1. Study Area

The forest area was situated in Barão de Cotegipe (27°37′15″ S and 52°22′47″ W, average 765 m a.s.l.), RS, Brazil. *Coccocypselum lanceolatum* plants were found covering soil patches (Figure S1) of the anthropized forest with *Araucaria angustifolia* as the dominant species, which was followed by other tall tree species such as *Podocarpus lambertii*, *Ilex paraguariensis*, *Ocotea* spp., etc. (Atlantic Forest biome). In this region, the predominant vegetative growth of *C. lanceolatum* was observed from May to September, blooming from November to January, and fruiting from March to April. The forest area is characterized by transitional vegetation, where remnants of mixed ombrophilous forest struggle to survive in an environment highly influenced by humans [34]. This is due to intensive agriculture in neighboring areas or by the intensive extraction of precious lambert species and yerba-mate for industry use. In this particular area, yerba-mate seedings had been replanted in the forest understory [35]. The climate is defined as subtropical humid (Cfa) using Köppen’s classification [36] with regularly distributed rainfall during the year (>1600 mm) and an average temperature > 22 °C in the hottest month. The soil is classified as Rhodic Haplodux, which is an acid soil of low natural fertility [37]. Some native plants from tropical regions, such as *Coccocypselum* spp., have evolved survival strategies to deal with high Al saturation in acidic and nutrient-poor soils [38].

### 2.2. Microclimate Measurements

Light intensity and quality were measured every two months during a one-year period at the surface of the soil in the forest where patches of *C. lanceolatum* were found. As an agrometeorological reference, sensors were also installed at 2 m above soil at a neighboring open area covered with short cut grassland. The light intensity was measured using a LI-190R quantum sensor (LI-COR Environmental, Lincoln, NE, USA), which measures photosynthetic photon flux density (PPFD) at 400–700 nm (μmol photons m^−2^ s^−1^). The light quality was expressed as the R:FR ratio, obtained with a SKR 110 sensor (Skye Instruments Ltd., Powys, UK), that measures radiation at 655–665 nm and 725–735 nm. The PPFD and R:FR were registered on an LI-1400 datalogger (LI-COR Environmental, Lincoln, NE, USA) by integrating sequential 10 s measurements into 10 min values. The light conditions of both environments were observed for two days from 10:00 h until 15:00 h in the same periods when leaf gas exchange measurements were performed.

### 2.3. Leaf Gas Exchange and Light Curve (A_net_—PPFD Curve) Measurements

The PPFD at the leaf scale, air temperature (T_air_, °C), leaf net CO_2_ assimilation (*A*_net_, µmol CO_2_ m^−2^ s^−1^), stomatal conductance (*g_s_*, mol H_2_O m^−2^ s^−1^), leaf transpiration (*E*, mmol H_2_O m^−2^ s^−1^), and intrinsic water use efficiency (iWUE = *A*/*g_s_*, µmol mol^−1^) were measured in situ with an LCpro Photosynthesis System (ADC Bioscientific Ltd., Hoddesdon, UK), using a broad chamber (6 cm^2^). Leaf gas exchange measurements were conducted every two months over a one-year period (from July of year 1 to May of year 2), which resulted in six temporal data sets. The measurements were performed between 10:00 h and 15:00 h, during the period of the highest diurnal assimilation, with a natural variation in air temperature (ranging between 9.4 and 35.2 °C depending on the measuring period), CO_2_ concentration of 402 ± 2.36 μmol mol^−1^ and relative humidity ranging between 38.9 and 61.1% depending on the measurement period.

*A*_net_–PPFD curves were constructed in September (early spring) during the vegetative growth phase, using the same device that was used for leaf gas exchange measurements. PPFD generated by a blue and red led light source varied in the following order: 1740, 1305, 870, 609, 348, 184, 87, 43, 17 and 0 μmol m^−2^ s^−1^, which was similar to that used for vegetation-type responses of the Cerrado plants, including *C. lanceolatum* [24]. The air CO_2_ concentration was maintained at ~400 mmol mol^−1^, and the leaf temperature was approximately 25 °C. An initial acclimation of 20 min was performed at 1740 µmol m^−2^ s^−1^, because this time has been sufficient for photosynthetic induction over increased photosynthetic CO_2_ assimilation from shade to high light levels in various C3 species [39]. Other measurements were taken after 7 min of acclimatization at each PPFD level. *A*_net_–PPFD curves were fitted using a nonrectangular hyperbola model [40] as shown in Equation (1):

(1) $$A_{net}=\frac{\Phi \cdot \mathrm{PPFD}+A_{\max\_\mathrm{gross}}-\sqrt{{\{⟦\Phi \cdot \mathrm{PPFD}+A_{\max\_\mathrm{gross}}⟧}^{2}-4\cdot \Theta \cdot \Phi \cdot A_{\max\_\mathrm{gross}}\}}}{2\cdot \Theta }-R_{d}$$

where *A*_net_ is the leaf net CO_2_ assimilation (μmol m^−2^ s^−1^), Φ is the apparent quantum yield (μmol μmol^−1^) calculated as the angle of the initial linear region of the *A*_net_–PPFD curves, *A*_max_gross_ is the gross photosynthesis under light saturation (μmol m^−2^ s^−1^), *R*_d_ is the dark respiration (μmol m^−2^ s^−1^), and θ is the curve convexity (dimensionless). The light compensation point (LCP, µmol m^−2^ s^−1^), maximum photosynthesis under light saturation (*A*_max_, μmol m^−2^ s^−1^, estimated as *A*_max_ = *A*_max_gross_ × 0.9 − *R*_d_), and light saturation point (LSP, µmol m^−2^ s^−1^, estimated as PPFD level for *A*_max_) were calculated from Equation (1).

Two age groups of leaves were evaluated: (a) young leaves, almost fully expanded with green–yellowish–violet coloration, which were located on the upper part of shoots and (b) adult leaves, which were fully expanded and characterized by dark green coloration (Figure S1). Both types were observed at two-monthly intervals with one repetition of the two leaf ages on individual plants. This procedure was performed for twelve plants at each measurement period for leaf gas exchange (n = 12), while for *A*_net_–PPFD curves, only three plants were used.

### 2.4. Statistical Analysis

R statistical software (version 4.2.1, R Core Team, Viena, Austria, 2024) was used to perform all analyses. One-way ANOVAs were performed to estimate the variance of parameters calculated from *A*_net_–PPFD curves, while two-way ANOVAs were used for (a) leaf gas exchange parameters (*A*_net_, *g_s_*, *E* and iWUE), PPFD and T_air_ at leaf scale, and (b) PPFD and R:FR where data were collected at 10-min intervals. A complete randomized design was used to assess the effects of (a) leaf age (young and adult leaves) and one-year dynamics (six measurement periods) or (b) environmental light (open area and forest soil) and one-year dynamics (six measurement periods). ANOVAs considered linear models (‘nlme’ package), where maximum likelihood was used to assess the significance of studied variables. The Bartlett homogeneity and the Shapiro normality tests were performed for each variable during each period. If the interaction was not significant, the model reduction was applied, and the model was refitted. The results are presented graphically as estimated means ± standard errors (SE), including *p*-values. The df, F and *p*-values are shown in the tables. All ANOVAs were performed at 95% confidence levels, which were followed by a Tukey honestly significant difference test (using ‘emmeans’ and ‘multcomp’ packages). The Pearson coefficient was used to correlate microclimate with leaf gas exchange parameters, using the complete matrix and the ‘corrplot’ package, separating three phenology phases (vegetative, blooming, and fruiting). A significance level of ≤0.05 was used for all analyses with an exception for the two situations where a marginal significance of <0.1 was discussed.

## 3. Results

### 3.1. Light Environment of Coccocypselum lanceolatum

**Figure 1:**
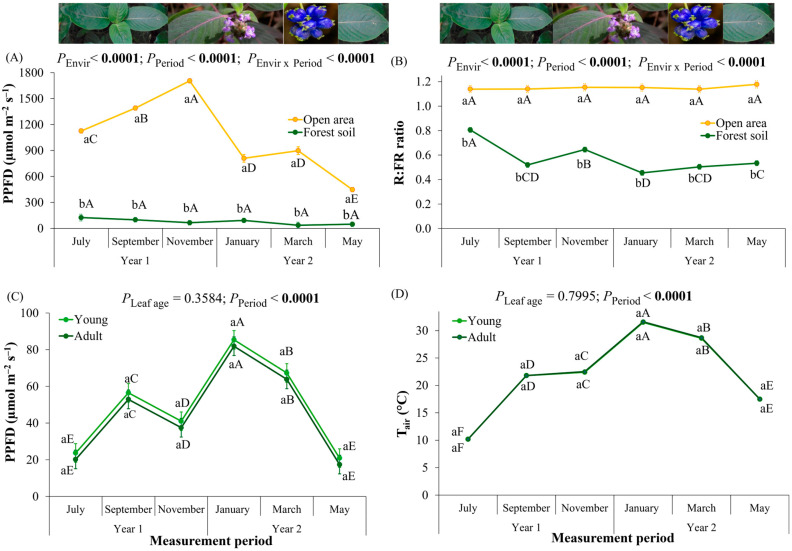
Microclimate during annual *Coccocypselum lanceolatum* phenology determinations over one year with measurement made every two months, starting in July and finishing in May of the following year: (**A**) photosynthetic photon flux density (PPFD) and (**B**) R:FR ratio for the diurnal period (10:00 h to 15:00 h) in an open area (at a height of 2 m) and from the forest soil surface (at a height of 0 m) (n = 62), (**C**) PPFD and (**D**) air temperature (T_air_) at the level of adult and young leaves (n = 12). Estimated mean ± SE and *p*-values (bold when significant) are also shown. Lowercase letters compare the responses of the two different environments or leaf ages for each measurement period, while uppercase letters compare the measurement periods for each environment or leaf age. Photos illustrating general phenological events are shown in the inserts (July, September, and May—vegetative growth, November to January—blooming and March—fruiting).

Light quantity (PPFD) was measured in the open area (at a height of 2 m), and it varied over the year but was relatively stable at the forest soil surface at a height of 0 m (Figure 1A, Table S1). Mean diurnal PPFD values (10:00 h to 15:00 h) were the highest in November in the open area (sunny days and elevated angle of the sun’s rays), while the lowest PPFD values were observed in May due to cloudy days during measurements and the lower angle of the sun’s rays (the end of autumn). The transmitted light reaching the forest soil surface was reduced to 4% (November and March) or 11% (July, January, and March) when compared to the open area. The R:FR ratio did not vary significantly in the open area (Figure 1B) despite significant PPFD variations (Figure 1A). The mean diurnal values for the R:FR ratio were 0.45 (January, closed forest canopy), 0.65 (November) and 0.81 (July, when many leaves dropped from the upper forest layers) (Figure 1B). The R:FR values of 0.15 were registered during two 10-min measurements in January.

The PPFD at leaf level (registered during leaf gas exchange measurements) showed significant temporal variations with the highest values seen in January and the lowest in July and May without significant differences between the two leaf ages (Figure 1C, Table S1). The PPFD at leaf scale showed variability that the sensor stationed on forest soil did not register (Figure 1A). T_air_ was the highest in January and March (summer) and the lowest in July (winter) and May (autumn) with no significant differences between leaf ages (Figure 1D).

### 3.2. Coccocypselum lanceolatum Leaf Gas Exchange Parameters

**Figure 2:**
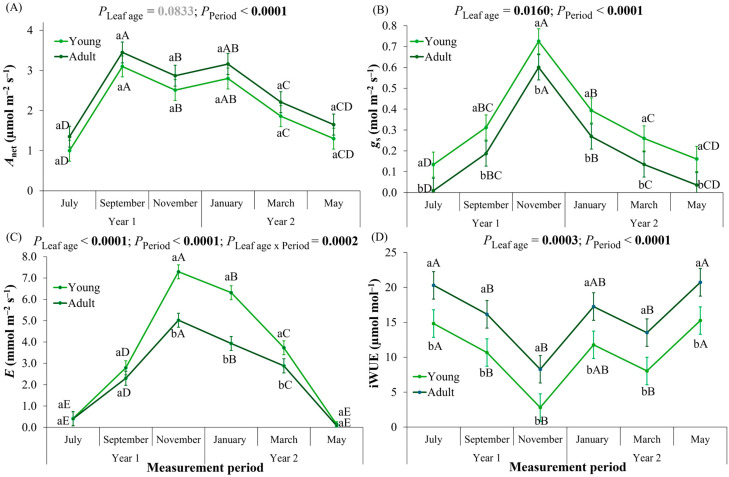
*Coccocypselum lanceolatum* leaf gas exchange for adult and young leaves over a one-year period measured every two months, starting in July and finishing in May of the following year: (**A**) leaf net CO_2_ assimilation (*A*_net_), (**B**) stomatal conductance (*g_s_*), (**C**) leaf transpiration (*E*), and (**D**) intrinsic water use efficiency (iWUE). Estimated mean ± SE and *p*-values (bold when significant, gray when marginally significant) are shown (n = 12). Lowercase letters compare responses of the two leaf ages for each measurement period, while uppercase letters compare the measurement periods for each leaf age.

All of the parameters for leaf gas exchange determinations varied over the periods of measurement and *C. lanceolatum* phenology (Figure 2, Table S1). They were additionally influenced by leaf age. A marginal effect of leaf age (*p* < 0.1) was observed only for *A*_net_ (Figure 2A). The adult leaves showed slightly higher *A*_net_ than young leaves (Figure 2A) and a higher iWUE over the whole one-year period (Figure 2D). On the other hand, *g_s_* and *E* were higher in young leaves, which lost more H_2_O than adult ones per leaf area (Figure 2B and 2C, respectively). Leaf *E* was not significantly different between the two leaf ages during the measurement periods when the lowest *A*_net_, *g_s_* and *E* were registered (May, July and September). The highest iWUE values (Figure 2D) were observed in May and July, periods of the lowest *g_s_* (Figure 2B), during predominantly vegetative stages. The lowest iWUE values were observed in November (Figure 2D) during intense blooming, when *g_s_* was the highest (Figure 2B), but not *A*_net_ (Figure 2A). Such iWUE responses over a one-year period suggested that this parameter was impacted more by water than by CO_2_ demand with differences seen between leaf ages (Figure 1D).

### 3.3. Photosynthetic Performance of Coccocypselum lanceolatum

**Figure 3:**
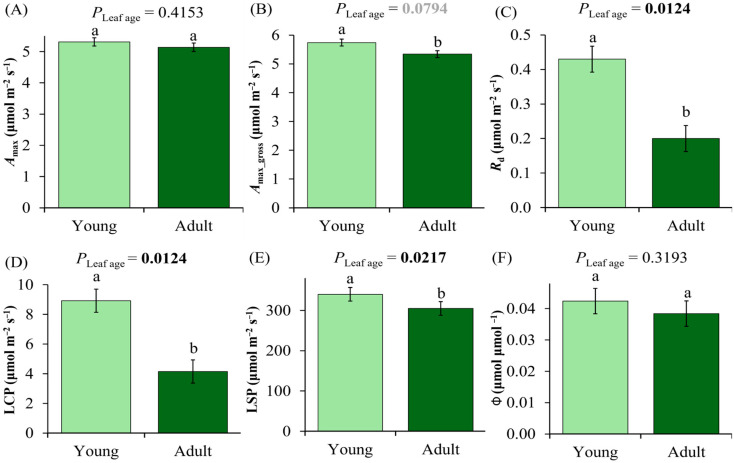
Parameters of photosynthetic responses to light (curves *A*_net—_PPFD) of *Coccocypselum lanceolatum* measured in September during vegetative stage on adult and young leaves: (**A**) maximum photosynthesis under saturating light (*A*_max_), (**B**) maximum gross photosynthesis under saturating light (*A*_max___gross_), (**C**) dark respiration (*R*_d_), (**D**) light compensation point (LCP), (**E**) light saturation point (LSP), and (**F**) apparent quantum yield (Φ). Estimated mean ± SE, and *p*-values (bold when significant, gray when marginally significant) are shown (n = 3). Lowercase letters compare responses of the two leaf ages.

The *A*_max___gross_ (Figure 3B, Table S2) was marginally different between young and adult leaves (*p* < 0.1), while *R*_d_, LCP, and LSP were highly different (Figure 3C–E, Table S2). Only for *A*_max_ and Φ (Figure 3A and 3F, respectively) was no statistical difference between the two leaf ages found. The *R*_d_ values were significantly higher in young leaves than in adult leaves (Figure 3C). As leaf age difference was not expressed in *A*_max_ (Figure 3A), the marginally significant differentiation in *A*_max___gross_ between two leaf ages (Figure 3B) can be explained by *R*_d_ variations.

*Coccocypselum lanceolatum* showed extremely low LCP with values of 8.9 and 4.2 µmol photons m^−2^ s^−1^ in young and adult leaves, respectively (Figure 3D). It is worth mentioning that the minimum PPFD at leaf level registered in this experiment was 6.77 µmol photons m^−2^ s^−1^, and that PPFD from 20 readings was approximately 10 µmol m^−2^ s^−1^ (all taken from July and May during the vegetative growth), which was associated with positive *A*_net_ values of 0.4–1.8 µmol CO_2_ m^−2^ s^−1^. Additionally, the lowest T_air_ was 9.3 °C, which was registered during the leaf gas exchange measurements in July during vegetative growth. Twenty-four T_air_ readings were approximately 10 °C, which were all registered in July (winter), and they were associated with positive *A*_net_ values of 0.7–1.8 µmol CO_2_ m^−2^ s^−1^. Additionally, *C. lanceolatum* attained saturation in photosynthesis (LSP) at low PPFD levels with values of 340 and 305 µmol photons m^−2^ s^−1^ in young and adult leaves, respectively (Figure 3E).

### 3.4. Correlations Among Leaf Gas Exchange and Microclimate Variables in Relation to Coccocypselum lanceolatum Phenology

**Figure 4:**
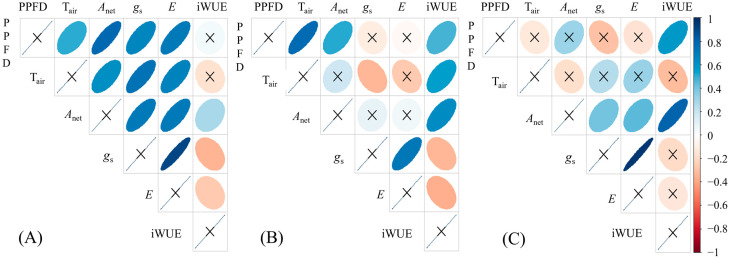
Graphical presentation of Pearson’s correlation coefficients (values corresponding to ellipse size and color intensities) with *p*-values (significant when ellipses are not crossed at <0.05) for correlations among: photosynthetic photon flux density (PPFD) at leaf scale, air temperature (T_air_), leaf net CO_2_ assimilation (*A*_net_), stomatal conductance (*g_s_*), leaf transpiration (*E*), and intrinsic water use efficiency (iWUE), during (**A**) vegetative growth (July, September and May, n = 72), (**B**) blooming (November and January, n = 48), and (**C**) fruiting (March, n = 24) of *Coccocypselum lanceolatum*. Data for both leaf ages were pooled for each phenological phase.

As microclimate variables changed together with *C. lanceolatum* phenology (Figure 1), the correlations among microclimate and leaf gas exchange parameters were performed for vegetative growth that occurred during July, September and May (Figure 4A), blooming that occurred during November and January (Figure 4B), and fruiting in March (Figure 4C). During vegetative growth, PPFD and T_air_ were positively correlated with all of the leaf gas exchange parameters observed here with the exception of iWUE (Figure 4A). In this phenophase, *A*_net_ was positively correlated with *g_s_*, *E* and iWUE, while *g_s_* and *E* were negatively correlated with iWUE. Such leaf gas exchange correlations were expected, although PPFD and T_air_ at the leaf level (Figure 1C and 1D, respectively) varied among the three observed measurement periods of vegetative growth. Due to the investments in reproductive organs during blooming and fruiting (Figure 4B and 4C, respectively), correlations among microclimate and leaf gas exchange parameters differed from the vegetative phenophase (Figure 4A). PPFD was positively correlated with *A*_net_ and iWUE, while T_air_ only correlated with iWUE during blooming (Figure 4B). In this phenophase, *g_s_* and *E* were negatively correlated with iWUE but positively correlated to each other. The lowest number of correlations was observed during fruiting (Figure 4C), when PPFD was positively correlated with *A*_net_ and iWUE (Figure 4C). During fruiting, *A*_net_ was positively correlated with *g_s_* and *E*; in the meantime, *g_s_* and *E* were positively correlated.

## 4. Discussion

The annual dynamics of *Coccocypselum lanceolatum* leaf gas exchange parameters were determined, being, to the best of our knowledge, the first ecophysiological report carried out during the different phenological phases of an herbaceous Rubiaceae (Figure 2). *Coccocypselum lanceolatum* showed shade tolerance through positive and high *A*_net_ and *g_s_* (Figure 2) relative to low light availability (Figure 1), which was supported by low iWUE, *R*_d_, LSP, and very low LCP values (Figure 3). 

The functional responses of *C. lanceolatum* were strongly driven by environment PPFD and T_air_ variations (energy factors) during vegetative growth, impacting on *A*_net_, *g_s_*, and *E*, while their direct impact was reduced during blooming and fruiting (Figure 4). During blooming, PPFD and T_air_ were positively associated with *A*_net_ and iWUE, while only iWUE was correlated to PPFD during fruiting. As energy factors and climate seasonality are known to drive the diversity pattern of Rubiaceae species [14], it was shown here that the impact of those environmental variables was modified by internal phenological phases of *C. lanceolatum* (Figure 4) or when environmental variables not observed here (such are relative humidity of air or soil moisture) became important.

As tropical forest communities share similar optimal temperatures for photosynthesis, independent of vegetative type and plant position in the canopy, photosynthetic performance seems to be optimized under ambient temperature regimes [41]. *Coccocypselum lanceolatum* showed tolerance to high and low temperatures, permitting physiological continuum even in autumn/winter PPFD ~10 µmol m^−2^ s^−1^ occurring at a T_air_ < 10 °C (Figure 2). Such responses suggested that some PIFs, similar to PIF7, could be involved in responses to shade and low temperatures of *C. lanceolatum*, which was analogous to responses to shade combined with warm temperatures of various tropical species [42]. This is one of the thermomorphogenetic mechanisms that could be studied in future works.

Shade-tolerant species could select leaf traits that promote efficient light energy capture while minimizing carbon losses under low light, expressed through low LCP, LSP, and *R*_d_, and increased water use efficiency under low light [43]. *Coccocypselum lanceolatum* showed very low LCP of 8.9 and 4.2 µmol m^−2^ s^−1^ in young and adult leaves, respectively (Figure 3D). Within the closed forest canopy of Cerrado savanna, where only adult leaves are observed in November (blooming), an LCP of 2 µmol m^−2^ s^−1^ was registered [24], which is even lower than in this paper (Figure 3D). This may be due to differences in phenological phase (vegetative versus blooming) or biome factors. The shade tolerance of some tropical Rubiaceae shrubs is explained by very low LCP [44]. The LSP in *C. lanceolatum* was 305 and 340 µmol photons m^−2^ s^−1^ in adult and young leaves, respectively (Figure 3E), indicating that leaves could efficiently use light up to such values due to the occurrence of sun flacks [45]. Photoinhibition occurs in shade leaves of shade-established *Psychotria rubra* (shrub Rubiaceae) when exposed to high light when a forest is cut down [46]. Such a reaction would probably happen after long exposure to elevated PPFD in *C. lanceolatum*, which is a response that could be studied in the future.

In the experiment that analyzed the Cerrado forest canopy, the *R*_d_ of understory herbaceous species is shown to be 1.97 µmol m^−2^ s^−1^ [24], which is much lower than in *C. lanceolatum* (0.43 and 0.20 µmol m^−2^ s^−1^ in young and adult leaves, respectively, Figure 3D). Also, in our results, *A*_max_ and Φ (~5 µmol m^−2^ s^−1^ and ~0.04 µmol µmol^−1^, respectively, Figure 3A and 3F) were two times lower than in Cerrado [24]. This again may be due to differences in phenological phases or biome factors. It is worth remembering that only three repetitions were used here for *A*_net_—PPFD curves, which may reduce the robustness of the parameters derived (Figure 3). As four parameters (*A*_max___gross_ *R*_d_, LCP, and LSP) showed significant differences between the two leaf ages, low repetition number was not a limiting factor. Additionally, our methodological *A*_net_–PPFD protocol followed the protocol performed for the Cerrado vegetative-type responses [24]. It is initiated with elevated light, which may induce photoinhibition or stomatal inhibition, potentially biasing *A*_max_ and Φ estimates downward.

Regarding leaf gas exchange dynamics in *C. lanceolatum*, deep shade adaptation was shown from relatively high *A*_net_ values of 3.45 and 3.16 μmol m^−2^ s^−1^ observed during vegetative growth (September) and blooming (January), respectively (Figure 2A). Such values were obtained under a PPFD of 50 μmol m^−2^ s^−1^ and T_air_ of 20.3 °C (September) and a PPFD of 80 μmol m^−2^ s^−1^ and T_air_ of 31.3 °C (January), respectively. An elevated T_air_ and relatively high PPFD registered in the forest understory in January (Figure 1C and 1D, respectively) induced similar *g_s_* as in September (Figure 2B), but it was much higher *E* in January than in September (Figure 2C). The light-induced opening of stomata is driven by two distinct wavelength regions: (1) the red region related to photosynthesis under elevated PPFD, as the main mechanism for coordinating stomatal movements with photosynthesis, and (2) blue light responses of the guard cells saturated at low PPFD, independent of photosynthesis, that are important for early morning stomatal opening [47]. Even the low R light reaching shaded leaves of *C. lanceolatum* (Figure 1A,B) was sufficient to regulate stomatal functionality (*g_s_*), which in turn regulated *A*_net_ and *E*. Such responses showed that this species successfully adjusted its functional responses even under R:FR from 0.8 to 0.15 (Figure 1B). Those values are considered low [48] or extremely low (R:FR < 0.2) [33]. Additionally, stomatal opening and *A*_net_ were strongly modified by high T_air_ during blooming (January), while during vegetative growth (May and July), the limiting factor was low T_air_. Such crossed responses again indicate the necessity of future research about thermomorphogenetic mechanisms of light and temperature perception by phytochromes [49] in this species.

All leaf gas exchange parameters differed between young and adult leaves in *C. lanceolatum* (Table S1). This is related to the fact that young leaves undergo a transition from resource acquisition to resource conservation during their developmental and growth processes [50]. The adult leaves showed higher *A*_net_ and iWUE than young ones (Figure 2A and 2D, respectively), while *g_s_* and *E* were lower in adult leaves than in young leaves (Figure 2B and 2C, respectively), as expected in tropical plant species [51]. The exception was seen in the interaction between leaf age and measurement period for *E* over vegetative growth (July, September and May) when two leaf ages showed similar *E* values (Figure 2C). Despite the young leaves being sinks, leaves of two ages in *C. lanceolatum* did not differ when extreme *A*_net_ values were observed—the lowest and the highest (Figure 2A), indicating that the strategy of this species was focused on maximizing carbon (*A*_net_), especially in the vegetative stage, and not on economizing water (*E*) at both leaf ages.

As the literature about herbaceous species of Rubiaceae is unavailable, we compare leaf age responses of *C. lanceolatum* with certain understory tree and shrub species of this family. Shaded young and adult *Coffea arabica* leaves exhibit different photosynthetic behaviors and acclimation capabilities, with young leaves being significantly more vulnerable to light fluctuations than adult leaves, especially when sudden light increases occur [52]. This is explained by the efficient photoprotection related to the ability of adult leaves to export sucrose, preventing Calvin cycle discontinuity by sucrose accumulation. In the understory neotropical shrub *Psychotria limonensis*, young and adult leaves do not show changes in overall photochemical efficiency (F_v_/F_m_), and no relationship between photochemical efficiency and leaf temperature is observed, suggesting that leaves are susceptible to photoinhibition independently of their ontogenetic stage [23]. In future studies, photoprotection and sucrose metabolism should be investigated to understand mechanisms of functional responses of *C. lanceolatum* related to leaf age and ontogeny.

The presence of colleters [7], hairy leaves, and the position of abaxial stomata [6] could help *C. lanceolatum* to survive dry periods in the Brazilian Atlantic Rainforest and Cerrado and regulate gas exchange and water loss. For example, iWUE had an interval of 3–21 μmol mol^−1^ (Figure 2D), which are low values when compared to the deep-shade leaves of other C3 species such as *Theobroma cacao* [53], or *C. arabica* [19], with iWUE of 80–120, or 120–430 μmol mol^−1^, respectively. The increased rate of leaf-level iWUE occurs on a global scale in forest biomes (0.2 ± 0.02 μmol mol^−1^ year^−1^) with increased CO_2_ and reduced *g_s_* due to climate change [54]. On the other hand, *C. lanceolatum* displayed relatively high *g_s_*, from 0.01 to 0.73 mol m^−2^ s^−1^, compared with 0.02 to 0.10 mol m^−2^ s^−1^ in *T. cacao* [53] or 0.01 to 0.18 mol m^−2^ s^−1^ in *C. arabica* [19]. Such a stomatal control of CO_2_ assimilation rate (Figure 2A) induced low iWUE values in *C. lanceolatum* (Figure 2D).

Shrubs and herbaceous Rubiaceae are known for their medicinal and nutritional values for humans and livestock [55]. Their use is often restricted to local populations [56], but their productivity and physiological potential have not been investigated. As *C. lanceolatum* additionally represents a potential alternative species for fruit production, the ecophysiological responses over the one-year growth and development cycle shown here could help the inclusion of this species for garden and horticultural cultivation under deep shade, using the space below trees, acting as a sustainable soil cover.

## 5. Conclusions

All leaf gas exchange parameters varied over a one-year period, proving our first hypothesis. Additionally, they were correlated with energy factors (PPFD and T_air_), especially during vegetative growth. PPFD and T_air_ showed low correlations with leaf gas exchange parameters during blooming and fruiting. Seasonality and phenology (reproductive investments) also were drivers of leaf gas dynamics. In answer to the second hypothesis, as a deep shade forest species, *C. lanceolatum* displayed low iWUE values, being adapted to maximize carbon gain, prioritizing high *g_s_* without economizing water. *Coccocypselum lanceolatum* showed extremely low LCP, low *R*_d_, *A*_max_ and Φ as adaptational traits to low-light availability. Finally, a third hypothesis was also proved: young and adult leaves expressed differences in *A*_net_, *g_s_*, *E*, iWUE, *A*_max___gross_ *R*_d_, LCP, and LSP. Finally, the ecophysiological responses over a one-year period could help in species inclusion into garden and horticultural cultivation under deep shade.

## Data Availability

Data used for calculations and scripts will be made available by the author on request.

## Supplementary Materials

The following supporting information can be downloaded at https://www.mdpi.com/article/10.3390/biology15130994/s1: Figure S1. *Coccocypselum lanceolatum:* patches in the forest understory and one plant/stem (height of 10–15 cm) with the adult and young pairs of leaves; Table S1: Two-way ANOVA of different linear models adjustments for environment (Env) and period of measurements over a one-year phenology (Period) or leaf age (Leaf age) and period of measurements and their interaction (Env × Period or Leaf age × Period) effects on light intensity (photosynthetic photon flux density, PPFD measured at open area and forest soil or at leaf level), light quality (red-to-far-red ratio, R:FR), T_air_ and leaf gas exchange traits (leaf net CO_2_ assimilation, *A*_net_; stomatal conductance, *g_s_*; leaf transpiration, *E*; intrinsic water use efficiency, iWUE) of *Coccocypselum lanceolatum*; Table S2. One-way ANOVA for leaf age effects on performance parameters (maximum photosynthesis under saturating light (*A*_max_), maximum gross photosynthesis under saturating light (*A*_max___gross_), dark respiration (*R*_d_), light compensation point (LCP), light saturation point (LSP), and apparent quantum yield (Φ) derived from light response curves of *Coccocypselum lanceolatum*.

## Abbreviations

The following abbreviations are used in this manuscript:

*A*_net_	Leaf net CO_2_ assimilation
*A*_max_	Maximum photosynthesis under natural CO_2_ and saturating light conditions
*A*_max_gross_	Maximum gross photosynthesis under natural CO_2_ and saturating light conditions
*A*_net_—PPFD curves	Curves of leaf CO_2_ assimilation (*A*) to increasing photosynthetic photon flux density (PPFD)
*E*	Leaf transpiration
Φ	Apparent quantum yield efficiency
*g*_s_	Stomatal conductance
LCP	Light compensation point
LSP	Light saturation point
PPFD	Photosynthetic photon flux density
R:FR	Red-to-far-red ratio
*R*_d_	Dark respiration
iWUE	Intrinsic water use efficiency
