# Supplementary material for: One-Year Phenology of Leaf Gas Exchange Dynamics in Coccocypselum lanceolatum
Source: Biology (Basel). 2026 Jun 24;15(13):994. doi: 10.3390/biology15130994 (PMC13360093; doi:10.3390/biology15130994)
Supplement: Supplementary file 1 [file biology-15-00994-s001.zip › biology-4373068-supplementary.pdf]

Supplementary Material for Article

One-Year Phenology of Leaf Gas Exchange Dynamics in *Coccocypselum lanceolatum*

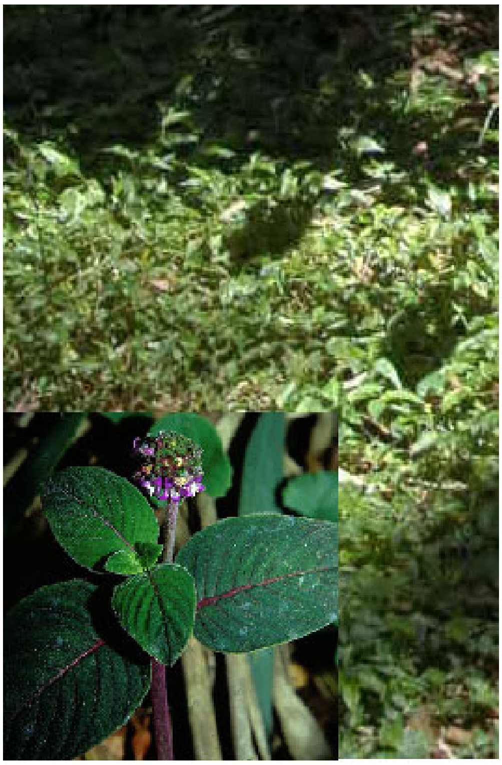

Figure S1. *Coccocypselum lanceolatum*: patches in the forest understory and one plant/stem (height of 10–15 cm) with the adult and young pairs of leaves.

Table S1. Two-way ANOVA* of different linear models adjustments for environment (Env) and period of measurements over a one-year phenology (Period) or leaf age (Leaf age) and period of measurements and their interaction (Env x Period or Leaf age x Period) effects on light intensity (photosynthetic photon flux density, PPFD measured at open area and forest soil or at leaf level), light quality (red-to-far-red ratio, R:FR), T_{air} and leaf gas exchange traits (leaf net CO_2 assimilation, A_{net}; stomatal conductance, g_s; leaf transpiration, E; intrinsic water use efficiency, iWUE) of *Coccocypselum lanceolatum*.

	Variable	Df Env	*F*-value Env	Df Period	*F*-value Period	Df Env x Period	*F*-value Env x Period	Df Residuals	*P(>F)* Env	*P(>F)* Period	*P(>F)* Env x Period
Figure 1	PPFD	1	4818.210	5	173.250	5	157.44	732	**< 0.0001**	**< 0.0001**	**< 0.0001**
	R:FR	1	2735.655	5	21.901	5	24.514	732	**< 0.0001**	**< 0.0001**	**< 0.0001**
	Variable	Df Leaf age	*F*-value Leaf age	Df Period	*F*-value Period	Df Leaf age x Period	*F*-value Leaf age x Period	Df Residuals	*P(>F)* Leaf age	*P(>F)* Period	*P(>F)* Leaf age x Period
	$PPFD_{Leaf\ level}$	1	0.8493	5	76937	-	-	137	0.3584	**< 0.0001**	-
	T_{air}	1	0.0648	5	1319.04	-	-	137	0.7995	**< 0.0001**	-
Figure 2	A_{net}	1	3.0437	5	11.6798	-	-	137	0.0832	**< 0.0001**	-
	g_s	1	5.9419	5	11.7995	-	-	137	**0.0161**	**< 0.0001**	-
	E	1	28.275	5	110.229	5	5.2266	132	**< 0.0001**	**< 0.0001**	**0.0002**
	iWUE	1	13.4863	5	6.4934	-	-	137	**0.0003**	**< 0.0001**	-

* Confidence level was 0.95. *P*-values marked in bold when significant, while the marginally significant effect was marked in gray.

Table S2. One-way ANOVA* for leaf age effects on performance parameters (maximum photosynthesis under saturating light (A_{max}), maximum gross photosynthesis under saturating light (A_{max_gross}), dark respiration (R_d), light compensation point (LCP), light saturation point (LSP), and apparent quantum yield (Φ) derived from light response curves of *Coccocypselum lanceolatum*.

Variable	Df	Df Residuals	*F*-value	*P(>F)*
A_{max}	1	4	0.8241	0.4153
A_{max_gross}	1	4	5.4732	**0.0794**
R_d	1	4	18.671	**0.0124**
LCP	1	4	18.671	**0.0124**
LSP	1	4	13.364	**0.0216**
Φ	1	4	1.2909	0.3193

* Confidence level was 0.95. *P*-values marked in bold when significant, while the marginally significant effect was marked in gray.
